# Nanomaterial Technology and Triple Negative Breast Cancer

**DOI:** 10.3389/fonc.2021.828810

**Published:** 2022-01-12

**Authors:** Kai Hou, Zeng Ning, Hongbo Chen, Yiping Wu

**Affiliations:** Department of Plastic and Cosmetic Surgery, Tongji Hospital, Tongji Medical College, Huazhong University of Science and Technology, Wuhan, China

**Keywords:** nanomaterial, nanotechnology, nanobiosensors, triple negative breast cancer, drug delivery, vaccines

## Abstract

Triple negative breast cancer (TNBC) is a malignant breast cancer subtype that is prone to progression, with high associated metastasis and five-year mortality rates and an overall poor prognosis. Chemotherapy is usually administered to treat TNBC without additional targeted therapies. Novel nanomaterials have a variety of excellent physical and chemical properties and biological functions (including targeting specificity), and contrast agents and drug delivery vectors based on nanotechnology are progressing towards a more accurate and targeted direction. This review discusses the mechanisms of action and prospects for the use of nanotechnology in the treatment of TNBC, thus providing potential new strategies for the diagnosis and treatment of TNBC.

## Introduction

Cancer is a major public health problem worldwide and the second leading cause of death in the United States. Cancer mortality rates have been rising throughout most of the 20^th^ and 21^st^ centuries, with 1,898,160 new cancer cases and 608,570 cancer deaths expected in the United States as of 2021 (1). From 2010 to 2016, the five-year overall survival rate for all diagnosed cancers in the United States was 67% (68% for Caucasians and 63% for African Americans) (1, 2). Prostate cancer (98%), melanoma (93%) and female breast cancer (90%) had the highest associated survival rates, while pancreatic cancer (10%), liver cancer (20%), esophageal cancer (20%) and lung cancer (21%) had the lowest survival rates (3, 4).

Improved cancer survival rates seen in recent decades are mainly due to advances in diagnosis and treatment, specifically reflecting advances in screening, diagnosis, and surgery (e.g., pathological staging, thoracoscopic surgery). Therapies for metastatic disease include targeted therapy (e.g., precision medicine), chemotherapy, radiotherapy, immunotherapy (e.g., programmed cell death protein-1 and programmed death ligand-1 inhibitors), and novel material-based therapies (1, 5–9). The disruptive effects of the coronavirus disease 2019 (COVID-19) pandemic on healthcare delivery include disruptive effects with respect to the diagnosis and treatment of cancer patients due to delays in diagnosis and treatment, reduced access to care, and delays or closures of healthcare facilities and systems. These factors are likely to result in short-term, spurious declines in cancer rates followed by an increase in advanced disease and associated cancer mortality rates (1, 10, 11).

## Breast Cancer

Women comprise 49.5 percent of the global population, and are disproportionately represented among elderly people over age 60. In fact, due to population growth and aging, the global cancer burden for women has been increasing in all countries regardless of income level. Breast cancer is the most common malignancy among women worldwide, with one study estimating that approximately 160,000 patients with advanced breast cancer were diagnosed in the United States as of 2017 (12).

Due to the influence of multi-modal factors, such as genetic susceptibility, lifestyle, and other environmental factors, breast cancer incidence and mortality show extreme variance across countries and demographics (13). For example, breast cancer incidence is higher in high-income regions (e.g., 92 per 100,000 in North America) as compared with low-income regions (e.g., 27 per 100,000 in Central Africa and East Asia) (14). However, many low-income and middle-income countries, including countries in sub-Saharan Africa and developing countries in Asia, have low breast cancer incidence rates due to delays in healthcare delivery, late diagnoses, and limited access to treatment due to low mammography coverage and limited overall treatment options (15).

At the molecular level, breast cancer molecular signatures include the activation of human epidermal growth factor receptor 2 (HER2, encoded by *ERBB2*), the activation of hormone receptors (estrogen receptors and progesterone receptors), and *BRCA* mutations (16, 17). Intrinsic classifications delineated in 2000 distinguish four breast cancer subtypes: Luminal A and Luminal B, HER2-enriched, and triple negative breast cancer (TNBC). This classification system shifts the clinical management of breast cancer from a cancer burden-based approach to a biologically-centered approach. Currently, clinical practice classifies five breast cancer subtypes (luminal A, luminal B, HER2-enriched (HER2+), basal-like and normal-like) based on histological and molecular characteristics, including TNBC. TNBC is defined according to the following criteria: estrogen receptor-negative (ER–), progesterone receptor-negative (PR–), HER2–, high grade, and high Ki67 index tumors, either NST (no special type) histology or special type histology (metaplastic, adenoid cystic, medullary-like, secretory), and a generally poor prognosis (18).

There are differences in prognosis among breast cancer subtypes. Approximately 10-15% of TNBC cases present with poor prognoses due to the lack of targeted therapy for TNBC, aside from chemotherapy (19, 20). Morphologically, approximately 90% of TNBC cases present as infiltrating ductal carcinoma, while the remaining cases are classified as apocrine carcinoma, lobular carcinoma, adenoid cystic carcinoma, and metaplastic carcinoma (21–23). There are six different TNBC subtypes, including basal like 1, basal like 2, mesenchymal like, mesenchymal stemlike, intracavitary androgen receptor, and immune regulation subtypes. TNBC heterogeneity clinically refers to different breast cancer subtypes presenting with a triple negative phenotype. Gene expression profiles and genetic outcomes for each class show differences, thus illustrating the inherent complexity of TNBC (21).

Triple-negative breast cancer is more likely to recur as compared with other breast cancer subtypes, with five-year specific survival rates of 85% for stage 3 triple-negative cancer, as compared with survival rates ranging from 94-99% for hormone receptor-positive and ERBB2-positive cancers (24). The distribution of breast cancer molecular subtypes varies by race, with African and African American women having the highest rates of TNBC. TNBC presents with a higher rate of metastasis and the highest proportion of poorly differentiated or undifferentiated grades among all subtypes. These factors result in reduced survival rates (25).

## Breast Cancer Diagnosis and Treatment

Therapeutic strategies for treating breast cancer include local therapies (e.g., surgery, radiotherapy) as well as systemic therapies. Molecular subtypes have a profound influence on the therapeutic strategies selected for breast cancer. Specifically, systemic therapies have been developed primarily on the basis of molecular characteristics, including targeted chemotherapy, endocrine therapy for hormone receptor-positive diseases, anti-HER2 therapy for HER2-positive diseases, polymerase inhibitors for *BRCA* mutation carriers, and novel immunotherapy modalities (18).

In addition to surgical topical treatment, patients with hormone-receptor-positive cancer need endocrine therapy. A few of these patients also receive chemotherapy. Patients with ERBB2-positive cancer are treated with ERBB2-targeted antibodies or small molecule inhibitors combined with chemotherapy. Patients with triple-negative cancer typically receive only chemotherapy or radiation. Whether breast cancer patients receive radiotherapy is determined according to their specific indications. More and more patients are choosing to undergo systemic treatments, including targeted preoperative chemotherapy (i.e., neoadjuvant chemotherapy) following preoperative puncture examination (24). Breast cancer treatments based on nanotechnology are the focus of the current review.

## Nanotechnology and Cancer

Advances in nanotechnology over the past two decades offer potential new strategies for treating various diseases (26–32). Nanotechnology-based contrast agents and drug delivery vectors for disease diagnosis and treatment are progressing towards a more accurate and targeted direction. Currently, nanocarriers are mainly comprised of polymers, metals, lipids, nucleic acids, and proteins, including nanoparticles/tubes, micelles, dendrimers and liposomes (33). These smart nanoparticles can encapsulate drugs or probes and are coated with specially modified ligands that bind to receptors expressed at cell sites and ultimately affect cell function for the accurate and effective diagnosis and treatment of disease (34, 35).

Nanooncology is a branch of nanomedicine. Cancer diagnosis and treatment based on nanotechnology has received wide attention on a worldwide scale in the past decades (36–40). For example, in diagnostics, some nanoparticles have been developed into biomolecular vectors that can detect cancer biomarkers and play an important role in assisting cancer detection and monitoring cancer biomarkers, including proteins, antibody fragments, DNA fragments, and RNA fragments (37). For example, nanobiosensors are very sensitive and can detect multiple protein biomarkers within seconds (41, 42). Additionally, nanotechnology assisted molecular diagnostic technology has been increasingly implemented in imaging applications, which is conducive to the identification of cancer at an earlier and more accurate stage (43, 44).

In terms of treatment, nanotechnology has unique physicochemical properties, including a high surface volume ratio. In recent years, drug delivery systems based on nanomaterials, including micelles, nanoemulsions and liposomes, have been widely used. Nanomaterials can bind and load bioactive molecules, including DNA, RNA, drugs, and proteins. These bioactive molecules can easily cross many biological barriers and can easily be transported to the target. Therefore, nanomaterials are widely used in the loading and delivery of drugs for treating various cancers so as to improve the efficacy of chemotherapy combined with radiotherapy and photodynamic cancer therapy (7, 25, 45–47). To achieve complete tumor eradication, therapeutic agents need to be infused at extremely high levels. Moreover, within cancer immunotherapy using nanotechnology, nanoparticles carry T cells or natural killer cells and higher concentrations of anti-cancer drugs, achieving a strong and lasting anticancer immune response due to low concentrations of immune regulators (48, 49). Advances in nanotechnology, including virus-like sizes and high surface-volume ratios and surfaces that can be modified to precisely target specific cell types can be widely used in designing cancer vaccines (50).

Additionally, the cancer microenvironment plays a critical role in determining cancer survival and reducing mortality. Microenvironmental factors, such as cancer hypoxia or hyperglycemia and inflammation, are also directly associated with the survival and expansion of cancer cells. Interventions aimed at changing the microenvironment of cancer cells can induce cancer cell death and form the basis for new anticancer therapies (51).

## Nanotechnology and TNBC

TNBC is an important and recalcitrant breast cancer subtype. The treatment of patients with TNBC remains an immense clinical challenge, characterized by aggressive progression, high metastasis rates, and poor overall prognoses (52). Because standard endocrine therapy (i.e., HER2 targeting therapy) does not affect TNBC, anthracycline-based drugs and taxane chemotherapy are major means to achieving TNBC systemic treatment. These treatment modalities are highly effective. However, many cycles of chemotherapy and high doses of cytotoxic drugs employed to destroy cancer cells are likewise toxic to nearby healthy cells, causing adverse systemic effects such as hair loss, gastrointestinal symptoms, and thrombocytopenia (53, 54).

Moreover, chemotherapy resistance caused by P-glycoprotein overexpression, DNA damage repair, topoisomerase II mutations, low solubility and bioavailability of chemotherapy drugs, and the immune escape of cancer cells limits the therapeutic effects of drugs on TNBC. Chemotherapy resistance results in a recurrence rate of 50% and a mortality rate of 37% for TNBC (55, 56). Invasive proliferation, heterogeneity, and cancer resistance to therapeutic drugs are extreme challenges in the treatment metastatic breast cancer, which mainly metastasizes to local lymph nodes, bones, and the lungs (57).

Therefore, in order to avoid non-specific targeting and chemotherapy side effects among TNBC patients, the need to open up new molecular targets and treatments is much more urgent than for other types of breast cancer. Nanotechnology-based drug delivery systems are auspicious tools that can selectively target tumors and eliminate the cytotoxicity of drugs to other organs (58).

### Drug Delivery Systems for Nanotechnology

Nanodrug delivery systems mostly rely on enhanced permeation and retention effects for targeting drug delivery (59). In general, nanoparticles that can be applied to cancer treatment within nanoscience have the following physiochemical properties: tailored size and conformation, appropriate encapsulation capacity, high adhesion to the cancer environment, selective localization, enhanced cancer internalization through endocytosis, sustained and controllable drug release, a long cyclic half-life, minimal systemic toxicity, and safe biological elimination (60, 61). Research on the high expression of cancer targets and ligands *via* nanomaterials combined with other therapies (such as photodynamic therapy, chemotherapy, and radiotherapy) to produce therapeutic synergistic effects will be the key to the application of nanomaterials within TNBC.

Photothermal therapy (PTT) is ineffective in the treatment of TNBC due to the lack of effective therapeutic targets. In order to solve this problem, Cheng et al., used gold nanocage (AuNC) as a photothermal conversion agent combined with anti-heat shock protein monoclonal antibody (cmHSP) as a target ligand in order to prepare a microwave triggered heat shock protein (HSP)-tar gold nanosystem (CMHSP-AUNC). Microwave irradiation can effectively activate HSP70 overexpression in TNBC, thus meaningfully improving the targeting ability, accumulation in cancer area, and anti-cancer efficacy of CMHSP-AUNC (62). Xu et al. developed a nanoemulsion formulation with high stability for the systematic delivery of puerarin nanoPue. This modality reshapes the stromal microenvironment through nanoparticle treatment, down-regulates intracanceral reactive oxygen species (ROS) and oxidative stress, meaningfully reduces the connective tissue formation response within different types of solid tumors and enables nanoparticles to infiltrate more effectively into cancer parenchyma. Simultaneously, nanoPue, a powerful tumor microenvironment (TME) modulator, meaningfully improves the cancer immune microenvironment as well as the therapeutic efficiency of α-PD-L1 in TNBC models (63). Bhattacharya et al. have also developed thyquinone (TQ)-loaded hyaluronic acid (HA) coupled with Pluronic ^®^ P123 and F127 copolymer nanoparticles (HA-TQ-NPS) as selective drug carriers to deliver anticancer phytochemical TQ to TNBC cells. HA-TQ-NPS meaningfully promotes apoptosis, anti-metastasis, and anti-angiogenesis in TNBC cells *via* upregulation of microRNA-361 with no associated toxicity to normal cells (64).

### Nanomaterials as Adjuvant Immunotherapy for TNBC

The immune microenvironment affects the occurrence and development of breast cancer according to the principles of immune monitoring and immune editing. In the early stages of tumorigenesis, the immune microenvironment plays an anti-cancer role mainly through the cytokine environment (i.e., activated CD8+ and CD4+ T cells). In contrast, once the cancer becomes aggressive, the cellular composition of the microenvironment, including fibroblasts and the cytokine content associated with cancer, facilitates cancer promotion and is invaded by breast cancer cells (63).

Using immune cells within targeted cancer therapy is in line with the concept of using internal mechanisms within the host immune system to fight cancer. In this study, Prof. James Allison and Prof. Tasuku Honjo, the winners of the 2018 Nobel Prize in Medicine, investigated the use of immune checkpoint blockades in cancer treatment *via* inhibiting negative immune regulation. Immunotherapy has achieved some success thus far, thereby providing a new therapeutic strategy for TNBC treatment. Current immunotherapies include immune checkpoint blockers, cytotoxic T lymphocyte (CTL) exchange activation, adaptive cell transfer therapy (ACT), and TME regulation. Nanotechnology presents a novel immunomodulatory strategy that can be implemented as a personalized immunotherapy modality for TNBC (65).

Nanotechnology provides efficient and intelligent nanodelivery systems that facilitate the delivery of immune-stimulating adjuvants and cancer antigens to enhance antigen presentation and immunity and aid in the treatment of metastases. Nanoparticle carriers improve the solubility and bioavailability of immunotherapy modalities, including protection from degradation, thereby increasing therapeutic efficacy. Currently, nanoparticles (NPs) are already implemented to help improve antigen expression pathways by delivering epigenetic regulators and immune-stimulating cytokines (66). For example, Tran et al. evaluated different polyethylene-oxidation-polylactic acid (PEO-PLA) copolymer micelles, with vorinostat (HDACi) demonstrating better biosolubility, an increased half-life, and improved pharmacokinetics as compared with other modalities (67). NP-carrying bevacizumab and CRLX101 likewise showed good efficacy in TNBC treatment (68). Sulforaphane (SFN) downregulates histone deacetylase (HDAC6) mediated phosphatase and inhibits MDA-MB-231 and MDA-MB-468 cells. The expression of tensin homolog (PTEN) induces autophagy, meaningfully increasing the sensitivity of TNBC to doxorubicin (DOX). Thus, inhibition of cancer growth *via* autophagy induction due to SFN combined with therapeutic DOX may provide an effective approach for TNBC treatment (69). Although targeted nanodrugs have good potential, due to the biological distributions, pharmacokinetics, targeted population biodegradability, immunogenicity, and the complexity of dosing system design for nanodrugs, only a portion of nanodrug systems (e.g., polymer micelles, liposomes, nanoparticle couplings) progress to the clinical administration stage. More research is needed with respect to TNBC nanodrugs.

### Vaccines Based on Nanotechnology

Cancer vaccines are comprised of cancer cells and/or cancer antigens and lead to an effective anti-cancer host immune response. Cancer vaccines include DNA vaccines, Ab vaccines against idiotypic types and cancer-associated pathogens, and dendritic cell vaccines (70, 71). In Liu’s study, the researchers (including the authors of the current review) constructed nanoparticles to deliver an mRNA vaccine encoding the cancer-associated antigen MUC1 to dendritic cells (DCs) in lymph nodes, thus activating cancer-specific T cells. In this study, combining a simple mRNA vaccine with an anti-CTLA-4 monoclonal antibody meaningfully enhanced the anti-cancer host immune response and anti-cancer effects. These data support the use NP as messenger RNA vaccine vectors as well as the combined implementation of TNBC immunotherapies, NP-based messenger RNA vaccines, and CTLA-4 inhibitors (52).

## Conclusions

Triple negative breast cancer is a difficult and often intractable disease because of its high heterogeneity and low associated survival rates. Currently, the available treatment methods for patients diagnosed with TNBC are limited, especially with respect to refractory TNBC. Novel nanotechnology modalities represent auspicious strategies for efficient and accurate diagnoses and targeted therapies for TNBC due to their tailored physical and chemical properties and biological functions. Our work guides and informs future research directions and will ultimately contribute to informing medical guidelines.

## Author Contributions

All authors contributed to the design of the study and writing of the manuscript. KH and HC undertook the research, YW and ZN wrote the main manuscript text and prepared figures. YW revised the article critically for important intellectual content and final approval of the version to be submitted. All authors contributed to the article and approved the submitted version.

## Conflict of Interest

The authors declare that the research was conducted in the absence of any commercial or financial relationships that could be construed as a potential conflict of interest.

## Publisher’s Note

All claims expressed in this article are solely those of the authors and do not necessarily represent those of their affiliated organizations, or those of the publisher, the editors and the reviewers. Any product that may be evaluated in this article, or claim that may be made by its manufacturer, is not guaranteed or endorsed by the publisher.

## Abbreviations

COVID-19, coronavirus disease 2019; HER2, human epidermal growth factor receptor 2; ER, estrogen receptors; PR, progesterone receptors; TNBC, triple negative breast cancer; BL1, basal like 1; BL2, basal like 2; ML, mesenchymal like; MSL, mesenchymal stemlike; LAR, intracavitary androgen receptor; IM, immune regulation; ROS, reactive oxygen species; TME, tumor microenvironment; DOX, doxorubicin.

## References

[1] Siegel RL Miller KD Fuchs HE Jemal A. Cancer Statistics, 2021. CA Cancer J Clin (2021) 71(1):7–33. doi: 10.3322/caac.21654 33433946

[2] Sung H Ferlay J Siegel RL Laversanne M Soerjomataram I Jemal A. Global Cancer Statistics 2020: GLOBOCAN Estimates of Incidence and Mortality Worldwide for 36 Cancers in 185 Countries. CA Cancer J Clin (2021) 71(3):209–49. doi: 10.3322/caac.21660 33538338

[3] American Cancer Society Cancer Statistics 2021 Report. J Nucl Med (2021) 62(3):12N. 33622967

[4] Ferlay J Colombet M Soerjomataram I Parkin DM Pineros M Znaor A. Cancer Statistics for the Year 2020: An Overview. Int J Cancer (2021). doi: 10.1002/ijc.33588 33818764

[5] Malhotra J Jabbour SK Aisner J. Current State of Immunotherapy for Non-Small Cell Lung Cancer. Transl Lung Cancer Res (2017) 6(2):196–211. doi: 10.21037/tlcr.2017.03.01 28529902 PMC5420529

[6] Zeng W Jiang D Liu Z Suo W Wang Z Zhu D. An Injectable Hydrogel for Enhanced FeGA-Based Chemodynamic Therapy by Increasing Intracellular Acidity. Front Oncol (2021) 11:750855. doi: 10.3389/fonc.2021.750855 34631588 PMC8492932

[7] Suo M Liu Z Tang W Guo J Jiang W Liu Y. Development of a Novel Oxidative Stress-Amplifying Nanocomposite Capable of Supplying Intratumoral H2O2 and O2 for Enhanced Chemodynamic Therapy and Radiotherapy in Patient-Derived Xenograft (PDX) Models. Nanoscale (2020) 12(45):23259–65. doi: 10.1039/d0nr06594c 33206098

[8] Li Y Appius A Pattipaka T Feyereislova A Cassidy A Ganti AK. Real-World Management of Patients With Epidermal Growth Factor Receptor (EGFR) Mutation-Positive Non-Small-Cell Lung Cancer in the USA. PloS One (2019) 14(1):e0209709. doi: 10.1371/journal.pone.0209709 30608948 PMC6319739

[9] Horn L Spigel DR Vokes EE Holgado E Ready N Steins M. Nivolumab Versus Docetaxel in Previously Treated Patients With Advanced Non-Small-Cell Lung Cancer: Two-Year Outcomes From Two Randomized, Open-Label, Phase III Trials (CheckMate 017 and CheckMate 057). J Clin Oncol (2017) 35(35):3924–33. doi: 10.1200/JCO.2017.74.3062 PMC6075826 29023213

[10] Li J Gao R Wu G Wu X Liu Z Wang H. Clinical Characteristics of Emergency Surgery Patients Infected With Coronavirus Disease 2019 (COVID-19) Pneumonia in Wuhan, China. Surgery (2020) 168(3):398–403. doi: 10.1016/j.surg.2020.05.007 32675033 PMC7236668

[11] Li M Cheng B Zeng W Chen S Tu M Wu M. Analysis of the Risk Factors for Mortality in Adult COVID-19 Patients in Wuhan: A Multicenter Study. Front Med (Lausanne) (2020) 7:545. doi: 10.3389/fmed.2020.00545 32984387 PMC7479842

[12] Mariotto AB Etzioni R Hurlbert M Penberthy L Mayer M. Estimation of the Number of Women Living With Metastatic Breast Cancer in the United States. Cancer Epidemiol Biomarkers Prev (2017) 26(6):809–15. doi: 10.1158/1055-9965.EPI-16-0889 PMC5833304 28522448

[13] Ginsburg O Bray F Coleman MP Vanderpuye V Eniu A Kotha SR. The Global Burden of Women’s Cancers: A Grand Challenge in Global Health. Lancet (2017) 389(10071):847–60. doi: 10.1016/S0140-6736(16)31392-7 PMC6191029 27814965

[14] Torre LA Siegel RL Ward EM Jemal A. Global Cancer Incidence and Mortality Rates and Trends–An Update. Cancer Epidemiol Biomarkers Prev (2016) 25(1):16–27. doi: 10.1158/1055-9965.EPI-15-0578 26667886

[15] Allemani C Weir HK Carreira H Harewood R Spika D Wang XS. Global Surveillance of Cancer Survival 1995-2009: Analysis of Individual Data for 25,676,887 Patients From 279 Population-Based Registries in 67 Countries (CONCORD-2). Lancet (2015) 385(9972):977–1010. doi: 10.1016/S0140-6736(14)62038-9 25467588 PMC4588097

[16] Perou CM Sorlie T Eisen MB van de Rijn M Jeffrey SS Rees CA. Molecular Portraits of Human Breast Tumours. Nature (2000) 406(6797):747–52. doi: 10.1038/35021093 10963602

[17] N. Cancer Genome Atlas. Comprehensive Molecular Portraits of Human Breast Tumours. Nature (2012) 490(7418):61–70. doi: 10.1038/nature11412 23000897 PMC3465532

[18] Harbeck N Penault-Llorca F Cortes J Gnant M Houssami N Poortmans P. Breast Cancer. Nat Rev Dis Primers (2019) 5(1):66. doi: 10.1038/s41572-019-0111-2 31548545

[19] Salgado R Denkert C Demaria S Sirtaine N Klauschen F Pruneri G. The Evaluation of Tumor-Infiltrating Lymphocytes (TILs) in Breast Cancer: Recommendations by an International TILs Working Group 2014. Ann Oncol (2015) 26(2):259–71. doi: 10.1093/annonc/mdu450 PMC6267863 25214542

[20] Kojima YA Wang X Sun H Compton F Covinsky M Zhang S. Reproducible Evaluation of Tumor-Infiltrating Lymphocytes (TILs) Using the Recommendations of International TILs Working Group 2014. Ann Diagn Pathol (2018) 35:77–9. doi: 10.1016/j.anndiagpath.2018.05.007 29886396

[21] Mendes TF Kluskens LD Rodrigues LR. Triple Negative Breast Cancer: Nanosolutions for a Big Challenge. Adv Sci (Weinh) (2015) 2(11):1500053. doi: 10.1002/advs.201500053 27980912 PMC5115335

[22] Boyle P. Triple-Negative Breast Cancer: Epidemiological Considerations and Recommendations. Ann Oncol (2012) 23(Suppl 6):vi7–12. doi: 10.1093/annonc/mds187 23012306

[23] Gonzalez-Angulo AM Timms KM Liu S Chen H Litton JK Potter J. Incidence and Outcome of BRCA Mutations in Unselected Patients With Triple Receptor-Negative Breast Cancer. Clin Cancer Res (2011) 17(5):1082–9. doi: 10.1158/1078-0432.CCR-10-2560 PMC3048924 21233401

[24] Waks AG Winer EP. Breast Cancer Treatment: A Review. JAMA (2019) 321(3):288–300. doi: 10.1001/jama.2018.19323 30667505

[25] Zhu D Liu Z Li Y Huang Q Xia L Li K. Delivery of Manganese Carbonyl to the Tumor Microenvironment Using Tumor-Derived Exosomes for Cancer Gas Therapy and Low Dose Radiotherapy. Biomaterials (2021) 274:120894. doi: 10.1016/j.biomaterials.2021.120894 34029916

[26] Oliveira MB do Prado AH Bernegossi J Sato CS Lourenco Brunetti I Scarpa MV. Topical Application of Retinyl Palmitate-Loaded Nanotechnology-Based Drug Delivery Systems for the Treatment of Skin Aging. BioMed Res Int (2014) 2014:632570. doi: 10.1155/2014/632570 24772430 PMC3977527

[27] Saraiva C Praca C Ferreira R Santos T Ferreira L Bernardino L. Nanoparticle-Mediated Brain Drug Delivery: Overcoming Blood-Brain Barrier to Treat Neurodegenerative Diseases. J Control Release (2016) 235:34–47. doi: 10.1016/j.jconrel.2016.05.044 27208862

[28] Zhu XJ Feng JQ Zheng MZ Yang ZR Zhao L Zhang W. Metal-Protein Nanoparticles Facilitate Anti-VSV and H1N1 Viruses Through the Coordinative Actions on Innate Immune Responses and METTL14. Macromol Biosci (2021) 21(4):e2000382. doi: 10.1002/mabi.202000382 33522144

[29] Zhang Y Yang Z Xiang Y Xu R Zheng Y Lu Y. Evolutions of Antibiotic Resistance Genes (ARGs), Class 1 Integron-Integrase (Inti1) and Potential Hosts of ARGs During Sludge Anaerobic Digestion With the Iron Nanoparticles Addition. Sci Total Environ (2020) 724:138248. doi: 10.1016/j.scitotenv.2020.138248 32247117

[30] Lalotra AS Singh V Khurana B Agrawal S Shrestha S Arora D. A Comprehensive Review on Nanotechnology-Based Innovations in Topical Drug Delivery for the Treatment of Skin Cancer. Curr Pharm Des (2020) 26(44):5720–31. doi: 10.2174/1381612826666200819202821 32814523

[31] Greco SJ Rameshwar P. Mesenchymal Stem Cells in Drug/Gene Delivery: Implications for Cell Therapy. Ther Delivery (2012) 3(8):997–1004. doi: 10.4155/tde.12.69 22946432

[32] Liu Y Hong H Xue J Luo J Liu Q Chen X. Near-Infrared Radiation-Assisted Drug Delivery Nanoplatform to Realize Blood-Brain Barrier Crossing and Protection for Parkinsonian Therapy. ACS Appl Mater Interfaces (2021) 13(31):37746–60. doi: 10.1021/acsami.1c12675 34318658

[33] Kumari P Ghosh B Biswas S. Nanocarriers for Cancer-Targeted Drug Delivery. J Drug Target (2016) 24(3):179–91. doi: 10.3109/1061186X.2015.1051049 26061298

[34] Setyawati MI Kutty RV Leong DT. DNA Nanostructures Carrying Stoichiometrically Definable Antibodies. Small (2016) 12(40):5601–11. doi: 10.1002/smll.201601669 27571230

[35] Del Pilar Chantada-Vazquez M Lopez AC Vence MG Vazquez-Estevez S Acea-Nebril B Calatayud DG. Proteomic Investigation on Bio-Corona of Au, Ag and Fe Nanoparticles for the Discovery of Triple Negative Breast Cancer Serum Protein Biomarkers. J Proteomics (2020) 212:103581. doi: 10.1016/j.jprot.2019.103581 31731051

[36] Gao A Hu XL Saeed M Chen BF Li YP Yu HJ. Overview of Recent Advances in Liposomal Nanoparticle-Based Cancer Immunotherapy. Acta Pharmacol Sin (2019) 40(9):1129–37. doi: 10.1038/s41401-019-0281-1 PMC6786406 31371782

[37] Thambiraj S Hema S Shankaran DR. An Overview on Applications of Gold Nanoparticle for Early Diagnosis and Targeted Drug Delivery to Prostate Cancer. Recent Pat Nanotechnol (2018) 12(2):110–31. doi: 10.2174/1872210511666171101120157 29090672

[38] Song X Ruan L Zheng T Wei J Zhang J Lu H. A Reduction Active Theranostic Nanoparticle for Enhanced Near-Infrared Imaging and Phototherapy by Reducing Glutathione Level in Cancer Cells. J Nanosci Nanotechnol (2021) 21(12):5965–71. doi: 10.1166/jnn.2021.19514 34229792

[39] Zhang J Wang L You X Xian T Wu J Pang J. Nanoparticle Therapy for Prostate Cancer: Overview and Perspectives. Curr Top Med Chem (2019) 19(1):57–73. doi: 10.2174/1568026619666190125145836 30686255

[40] Pekkanen AM DeWitt MR Rylander MN. Nanoparticle Enhanced Optical Imaging and Phototherapy of Cancer. J BioMed Nanotechnol (2014) 10(9):1677–712. doi: 10.1166/jbn.2014.1988 25992437

[41] Chaturvedi VK Singh A Singh VK Singh MP. Cancer Nanotechnology: A New Revolution for Cancer Diagnosis and Therapy. Curr Drug Metab (2019) 20(6):416–29. doi: 10.2174/1389200219666180918111528 30227814

[42] Tran S DeGiovanni PJ Piel B Rai P. Cancer Nanomedicine: A Review of Recent Success in Drug Delivery. Clin Transl Med (2017) 6(1):44. doi: 10.1186/s40169-017-0175-0 29230567 PMC5725398

[43] Mansur AA Mansur HS Soriano-Araujo A Lobato ZI. Fluorescent Nanohybrids Based on Quantum Dot-Chitosan-Antibody as Potential Cancer Biomarkers. ACS Appl Mater Interfaces (2014) 6(14):11403–12. doi: 10.1021/am5019989 24956063

[44] Jayanthi V Das AB Saxena U. Recent Advances in Biosensor Development for the Detection of Cancer Biomarkers. Biosens Bioelectron (2017) 91:15–23. doi: 10.1016/j.bios.2016.12.014 27984706

[45] Karlsson J Vaughan HJ Green JJ. Biodegradable Polymeric Nanoparticles for Therapeutic Cancer Treatments. Annu Rev Chem Biomol Eng (2018) 9:105–27. doi: 10.1146/annurev-chembioeng-060817-084055 PMC6215694 29579402

[46] Kemp JA Shim MS Heo CY Kwon YJ. “Combo” Nanomedicine: Co-Delivery of Multi-Modal Therapeutics for Efficient, Targeted, and Safe Cancer Therapy. Adv Drug Delivery Rev (2016) 98:3–18. doi: 10.1016/j.addr.2015.10.019 26546465

[47] Chen D Chen C Huang C Chen T Liu Z. Injectable Hydrogel for NIR-II Photo-Thermal Tumor Therapy and Dihydroartemisinin-Mediated Chemodynamic Therapy. Front Chem (2020) 8:251. doi: 10.3389/fchem.2020.00251 32318547 PMC7154176

[48] Siegler EL Kim YJ Chen X Siriwon N Mac J Rohrs JA. Combination Cancer Therapy Using Chimeric Antigen Receptor-Engineered Natural Killer Cells as Drug Carriers. Mol Ther (2017) 25(12):2607–19. doi: 10.1016/j.ymthe.2017.08.010 PMC5768663 28919377

[49] Meir R Shamalov K Betzer O Motiei M Horovitz-Fried M Yehuda R. Nanomedicine for Cancer Immunotherapy: Tracking Cancer-Specific T-Cells in Vivo With Gold Nanoparticles and CT Imaging. ACS Nano (2015) 9(6):6363–72. doi: 10.1021/acsnano.5b01939 26039633

[50] Mi Y t. Hagan CT Vincent BG Wang AZ. Emerging Nano-/Microapproaches for Cancer Immunotherapy. Adv Sci (Weinh) (2019) 6(6):1801847. doi: 10.1002/advs.201801847 30937265 PMC6425500

[51] Vishwakarma M Piddini E. Outcompeting Cancer. Nat Rev Cancer (2020) 20(3):187–98. doi: 10.1038/s41568-019-0231-8 31932757

[52] Liu L Wang Y Miao L Liu Q Musetti S Li J. Combination Immunotherapy of MUC1 mRNA Nano-Vaccine and CTLA-4 Blockade Effectively Inhibits Growth of Triple Negative Breast Cancer. Mol Ther (2018) 26(1):45–55. doi: 10.1016/j.ymthe.2017.10.020 29258739 PMC5763160

[53] Shi Y Jin J Ji W Guan X. Therapeutic Landscape in Mutational Triple Negative Breast Cancer. Mol Cancer (2018) 17(1):99. doi: 10.1186/s12943-018-0850-9 30007403 PMC6046102

[54] Bayraktar S Gluck S. Molecularly Targeted Therapies for Metastatic Triple-Negative Breast Cancer. Breast Cancer Res Treat (2013) 138(1):21–35. doi: 10.1007/s10549-013-2421-5 23358903

[55] Sharma A Goyal AK Rath G. Recent Advances in Metal Nanoparticles in Cancer Therapy. J Drug Target (2018) 26(8):617–32. doi: 10.1080/1061186X.2017.1400553 29095640

[56] Denkert C Liedtke C Tutt A von Minckwitz G. Molecular Alterations in Triple-Negative Breast Cancer-the Road to New Treatment Strategies. Lancet (2017) 389(10087):2430–42. doi: 10.1016/S0140-6736(16)32454-0 27939063

[57] Gradishar WJ Moran MS Abraham J Aft R Agnese D Allison KH. NCCN Guidelines(R) Insights: Breast Cancer, Version 4.2021. J Natl Compr Canc Netw (2021) 19(5):484–93. doi: 10.6004/jnccn.2021.0023 34794122

[58] Thakur V Kutty RV. Recent Advances in Nanotheranostics for Triple Negative Breast Cancer Treatment. J Exp Clin Cancer Res (2019) 38(1):430. doi: 10.1186/s13046-019-1443-1 31661003 PMC6819447

[59] Wang Y Pei H Jia Y Liu J Li Z Ai K. Synergistic Tailoring of Electrostatic and Hydrophobic Interactions for Rapid and Specific Recognition of Lysophosphatidic Acid, an Early-Stage Ovarian Cancer Biomarker. J Am Chem Soc (2017) 139(33):11616–21. doi: 10.1021/jacs.7b06885 28782946

[60] Setyawati MI Kutty RV Tay CY Yuan X Xie J Leong DT. Novel Theranostic DNA Nanoscaffolds for the Simultaneous Detection and Killing of Escherichia Coli and Staphylococcus Aureus. ACS Appl Mater Interfaces (2014) 6(24):21822–31. doi: 10.1021/am502591c 24941440

[61] Wang J Sui L Huang J Miao L Nie Y Wang K. MoS2-Based Nanocomposites for Cancer Diagnosis and Therapy. Bioact Mater (2021) 6(11):4209–42. doi: 10.1016/j.bioactmat.2021.04.021 PMC8102209 33997503

[62] Cheng Y Bao D Chen X Wu Y Wei Y Wu Z. Microwave-Triggered/HSP-Targeted Gold Nano-System for Triple-Negative Breast Cancer Photothermal Therapy. Int J Pharm (2021) 593:120162. doi: 10.1016/j.ijpharm.2020.120162 33307159

[63] Xu H Hu M Liu M An S Guan K Wang M. Nano-Puerarin Regulates Tumor Microenvironment and Facilitates Chemo- and Immunotherapy in Murine Triple Negative Breast Cancer Model. Biomaterials (2020) 235:119769. doi: 10.1016/j.biomaterials.2020.119769 31986348 PMC7093100

[64] Bhattacharya S Ghosh A Maiti S Ahir M Debnath GH Gupta P. Delivery of Thymoquinone Through Hyaluronic Acid-Decorated Mixed Pluronic(R) Nanoparticles to Attenuate Angiogenesis and Metastasis of Triple-Negative Breast Cancer. J Control Release (2020) 322:357–74. doi: 10.1016/j.jconrel.2020.03.033 32243981

[65] Jia H Truica CI Wang B Wang Y Ren X Harvey HA. Immunotherapy for Triple-Negative Breast Cancer: Existing Challenges and Exciting Prospects. Drug Resist Update (2017) 32:1–15. doi: 10.1016/j.drup.2017.07.002 29145974

[66] Hu X Zhang J Xu B Jiang Z Ragaz J Tong Z. Multicenter Phase II Study of Apatinib, a Novel VEGFR Inhibitor in Heavily Pretreated Patients With Metastatic Triple-Negative Breast Cancer. Int J Cancer (2014) 135(8):1961–9. doi: 10.1002/ijc.28829 24604288

[67] Tran TH Ramasamy T Truong DH Shin BS Choi HG Yong CS. Development of Vorinostat-Loaded Solid Lipid Nanoparticles to Enhance Pharmacokinetics and Efficacy Against Multidrug-Resistant Cancer Cells. Pharm Res (2014) 31(8):1978–88. doi: 10.1007/s11095-014-1300-z 24562809

[68] Pham E Yin M Peters CG Lee CR Brown D Xu P. Preclinical Efficacy of Bevacizumab With CRLX101, an Investigational Nanoparticle-Drug Conjugate, in Treatment of Metastatic Triple-Negative Breast Cancer. Cancer Res (2016) 76(15):4493–503. doi: 10.1158/0008-5472.CAN-15-3435 27325647

[69] Yang F Wang F Liu Y Wang S Li X Huang Y. Sulforaphane Induces Autophagy by Inhibition of HDAC6-Mediated PTEN Activation in Triple Negative Breast Cancer Cells. Life Sci (2018) 213:149–57. doi: 10.1016/j.lfs.2018.10.034 30352240

[70] Postmus PE Kerr KM Oudkerk M Senan S Waller DA Vansteenkiste J. Early and Locally Advanced Non-Small-Cell Lung Cancer (NSCLC): ESMO Clinical Practice Guidelines for Diagnosis, Treatment and Follow-Up. Ann Oncol (2017) 28(suppl_4):iv1–iv21. doi: 10.1093/annonc/mdx222 28881918

[71] Vansteenkiste J De Ruysscher D Eberhardt WE Lim E Senan S Felip E. Early and Locally Advanced Non-Small-Cell Lung Cancer (NSCLC): ESMO Clinical Practice Guidelines for Diagnosis, Treatment and Follow-Up. Ann Oncol (2013) 24(Suppl 6):vi89–98. doi: 10.1093/annonc/mdt241 23860613

